# How do energy consumption, globalization, and income inequality affect environmental quality across growth regimes?

**DOI:** 10.1007/s11356-023-31797-7

**Published:** 2024-01-12

**Authors:** Abdurrahman Nazif Çatık, Çağla Bucak, Esra Ballı, Muge Manga, Mehmet Akif Destek

**Affiliations:** 1 Department of Economics, Faculty of Economics and Administrative Sciences, Ege University, Izmir, Turkey; 2 Department of Economics, Faculty of Economics and Administrative Sciences, Erzincan Binali Yıldırım University, Erzincan, Turkey; 3 Department of Economics, Gaziantep University, Gaziantep, Turkey; 4 Adnan Kassar School of Business, Lebanese American University, Beirut, Lebanon; 5 Research Methods Application Center of UNEC, Azerbaijan State University of Economics (UNEC), Baku, AZ1001 Azerbaijan

**Keywords:** Ecological footprint, EKC hypothesis, Panel threshold model, Renewable energy, Income inequality

## Abstract

This paper investigates the impacts of renewable and nonrenewable energy consumption, income inequality, and globalization on the ecological footprints of 49 countries for the period of 1995–2018. Panel cointegration test reveals a long-run relationship between the variables. Long-run parameter estimates derived from AMG and CCEMG, increasing income and nonrenewable energy consumption, have a significant positive impact on the ecological footprint, while countries that consume more renewable energy have seen an improvement in the quality of the environment. Conversely, neither income inequality nor globalization has a significant effect on national EFs. Evidence from the estimation of the panel threshold error correction model, where GDP growth is used as the transition variable, indicates a significant threshold effect, which supports a nonlinear relationship among the variables by identifying two distinct growth regimes: lower and upper. For the estimation sample, the positive and significant parameter estimates for economic growth in both growth regimes do not support the EKC hypothesis. The results indicate that renewable and nonrenewable energy consumption has a larger impact on the EF in the upper than lower growth regime. The threshold estimates are in line with the linear long-run estimates that do not indicate that income inequality has a significant impact on ecological footprint. However, globalization appears to negatively affect environmental quality in the lower growth regime.

## Introduction

The acceleration of global economic growth, coupled with the expansion of the population, advancements in transportation systems, increased reliance on fossil fuel consumption, and significant increases in greenhouse gas concentrations, has collectively contributed to the escalation of global temperatures and sea levels (Muneer et al. 2005; Asif and Muneer 2007; Olaganathan and Quigley 2017; Rehman et al. 2021; Tao et al. 2023). The rise in greenhouse gas concentrations, which are widely acknowledged as the leading contributors to the phenomenon of global warming, is predominantly attributed to carbon dioxide emissions and other associated pollutants. As a result, reducing greenhouse gas emissions is critical to keeping the global average temperature rise below the 2 °C limit as stipulated by the Paris Agreement (UNFCCC 2015).

In light of these environmental sustainability concerns, the analysis of the factors affecting environmental pollution has recently garnered considerable attention from scholars and policymakers. The main cause of rising environmental pollution is attributed to economic growth (Islam 2021). A number of studies have provided evidence that economic growth adversely affects the quality of environment (Alola et al. 2019; Miao et al. 2022). Furthermore, a substantial amount of research has been devoted to the investigation of the environmental Kuznets curve (EKC) hypothesis, which indicates that the connection between environmental degradation and economic growth follows an inverted U-shaped pattern. As a result, environmental degradation increases during the early stages of economic growth but then decreases as economic development progresses (Grossman and Krueger 1995).

Along with economic growth, the studies have focused on the effects of the various variables based on the objectives of the studies. For example, the majority of research has found that energy use has a considerable effect on environmental degradation (Balli et al. 2021; Rahman et al. 2021). It has been discovered that total energy consumption has a deteriorative impact on environmental quality, primarily due to a significant share of the energy mix being met through the use of fossil fuels. Conversely, several studies suggest that the use of renewable energy sources contributes substantially to the preservation of the environment and the promotion of sustainable development (Destek and Sinha 2020).

The empirical studies have not revealed conclusive results regarding other control variables. For example, there is a lack of consensus regarding the relationship between income inequality and environment. While some researchers find that income inequality increases environmental pollution (Jorgenson et al. 2017; Knight et al. 2017; Grunewald et al. 2017; Chen et al. 2020; Khan et al. 2022; Yang et al. 2022), others report that it reduces carbon emissions (Golley and Meng 2012; Jiao et al. 2021; Muhammad et al. 2022), and another group find no statistically significant relationship (Kasuga and Takaya 2017; Clement and Meunie 2010; Chen et al. 2020; Wu and Xie 2020; Zhao et al. 2021). Similarly, the literature on the effects of globalization has also yielded ambiguous results. For instance, Wang et al. (2020) and Saud et al. (2020) found a negative link between environmental problems and globalization, while Sabir and Gorus (2019), Pata (2021), and Destek et al. (2023a) found that globalization exacerbates environmental problems. Additionally, there are other studies that found an insignificant effect between environmental degradation and globalization, such as Ahmed et al. (2019).

The evidence on inconclusive results on the effects of variables on environmental pollution has led to two different directions on the estimation of the determinants of environmental degradation. First, preliminary research has examined the factors influencing CO_2_ emissions and other traditional pollutants such as SO_2_ and particulate matter. A recent focus has shifted from air pollution to the ecological footprint (EF), which includes not only air pollution but also natural resource consumption and waste generation. The EF has been widely recognized as a key indicator of sustainability due to its ability to quantify the multidimensional aspects of environmental quality, and policymakers have used it to monitor and evaluate progress toward sustainable development objectives (Pata 2021). Second, regarding the methodologies employed, numerous studies investigating the determinants of the EF have used linear models, which assume a stable and time-invariant relationship between the explanatory variables and the environmental indicators. However, constant parameter specification assumption may not hold when the relationships among the variables follow a nonlinear pattern, as indicated by the EKC hypothesis. Furthermore, the use of linear specification in the analysis of the EKC might pose serious econometric problems due to the possible near-perfect collinearity between the polynomials of GDP (Sirag et al. 2017). Given this background, another contribution of this study to the literature is to analyze the presence of the EKC and examine the other determinants of the EF by using the panel threshold model. This specification is favored over the alternatives due to its ability to discern the impacts of distinct regimes on the EF that are influenced by particular variables’ behavior, including economic growth and income inequality (Chen et al. 2022; Wang et al. 2023).

In light of the aforementioned research objectives, this investigation seeks to address the following questions: (1) Does the EKC hypothesis hold in terms of the EF of the countries? (2) What is the impact of the consumption of renewable and non-renewable energy sources on the EF under different growth regimes? (3) Do income inequality and globalization affect environmental quality across different growth regimes?

The remaining sections of the article are structured as follows. A concise literature review of EF determinants is presented in the second section. The data utilized in the study are described in the third section. The fourth section describes the methodology employed. The fifth section presents the empirical findings, including estimates of long-run parameters and the panel threshold error correction model. The fifth section concludes by discussing the policy implications of the findings.

## Overview of literature

### Economic growth and environment

The impact of economic growth on environmental pollution was first elaborated by Grossman and Krueger (1995). This pioneering study sparked the interest of scholars, leading to further research on its applicability to different countries using various methodologies. In particular, many studies examined the EKC theory, using CO_2_ emissions as a measure of pollution, with contradictory results. For example, some studies concluded that the EKC hypothesis is not valid (Akbostancı et al. 2009; Du et al. 2012; Chandran and Tang 2013; Al-Mulali et al. 2015a; Mikayilov et al. 2018), whereas others confirmed it (Apergis and Ozturk 2015; Dogan and Inglesi-Lotz 2017; Khan et al. 2019; Rahman et al. 2022).

Another group of studies examined the validity of the EKC hypothesis using the EF as an indicator. Ozturk et al. (2016) discovered support for the EKC theory in upper-middle and high-income countries, while Al-Mulali et al. (2015b) verified the validity of EKC hypothesis for these nations. Similarly, Charfeddine and Mrabet (2017) confirmed the EKC for 15 MENA countries, and Danish et al. (2020) observed its presence in BRICS countries. Conversely, Al-Mulali et al. (2015b) found no support for the EKC theory using the EF measure for low- and lower-middle-income countries, Ozturk et al. (2016) for low- and lower-middle-income countries, Mrabet et al. (2017) for Qatar, and Destek and Sinha (2020) for 24 OECD countries. The load capacity factor, computed by dividing biocapacity by EF, was considered another overall indicator of environmental degradation (Guloglu et al. 2023; Kartal and Pata 2023; Pata et al. 2023, 2024a; Caglar et al. 2023; Pata and Destek 2023; Ozcan et al. 2023). Additionally, several studies explored the association between economic growth and the environment without taking into account EKC theory. The majority of studies found that economic growth increases the EF (Alola et al. 2019; Danish and Wang 2019; Nathaniel and Khan 2020; Sharif et al. 2020; Ali et al. 2021; Usman et al. 2021; Yang et al. 2021; Çakmak and Acar 2022; Miao et al. 2022).

In terms of methodology, some studies tested the EKC hypothesis using threshold models with different threshold variables. According to Aye and Edoja (2017), the relationship between CO_2_ emissions and GDP follows a U-shaped pattern. Sirag et al. (2017) examined the EKC relationship between CO_2_ and GDP per capita for the countries with different income groups using the dynamic panel threshold model and did not find support for the EKC hypothesis. Furthermore, Aydin et al. (2019) found no support for the hypothesis based on their analyses of the link between EF and GDP for 26 EU member states using the PSTR model with GDP as the threshold variable.

In contrast, Wu and Liu (2020) used urbanization, openness, industrial structure, and energy efficiency as the threshold variables instead of GDP to test the hypothesis for China’s provinces. It was determined that there are statistically significant threshold effects associated with both industrial structure and urbanization on the inverted U-shaped relationship between the EF and GDP. Li and Li (2021) concluded that EKC holds true in high-income countries when the degree of aging serves as a transition variable. Simionescu (2021) also provided support for the hypothesis by demonstrating an N-shaped relationship between CCE countries’ GHG emissions and GDP data. Using urbanization as the threshold variable, Wang et al. (2022) showed that urbanization strengthens the positive association between the economic growth, CO_2_ emissions, and the EF in 134 countries. In addition, economic growth had a greater positive effect on the EF than carbon emissions when urbanization was considered the threshold variable. Wang et al. identified a correlation between economic growth and CO_2_ emissions that followed an N-shaped pattern (2023). Ullah et al. (2021) concluded that GDP has a positive impact on the EF for the world’s top 15 renewable energy consumption countries when utilizing renewable energy as a threshold variable. Similarly, Li et al. (2022a, b) found that, for the 10 most visited countries, GDP has a positive impact on the EF when utilizing tourism as a threshold variable. However, neither study made any comment on the validity of the EKC.

In summary, the varied results demonstrated the complexity of the relationship between environmental and economic development. While some studies supported the EKC hypothesis, others suggested alternative patterns or non-linear relationships. Further research and analysis are therefore needed to better understand these dynamics and their implications for sustainable development. There is also a lack of analysis to test the EKC using EF as a proxy for pollution, as only one study was found conducted on the EU sample by Aydin et al. (2019). However, given the global impact of the climate crisis, a larger country sample would increase the significance of the analysis results. This broader scope would allow for a more comprehensive discussion of policy implications.

### Income inequality and environment

As discussed earlier, the relationship between environmental degradation and inequality has become more prominent due to rising income inequality and the climate crisis. The existing body of empirical research extensively examines and raises inquiries regarding the connection between environmental pollution and inequality. Specifically, these studies examine the link between environmental degradation and inequality in various countries or regions within countries through the use of CO_2_ as an environmental indicator. Many studies found a positive association between income inequality and CO_2_ (Torras and Boyce 1998; Magnani 2000; Golley and Meng 2012; Baek and Gweisah 2013; Liu et al. 2019b; Padhan et al. 2019; Baloch et al. 2020), whereas others reported an adverse relationship (Heerink et al. 2001; Huang and Duan 2020; Wu and Xie 2020; Wan et al. 2022). Furthermore, it is notable that the conclusions within a single study can vary depending on the choice of variables or the dynamics of the period under consideration.

Hübler (2017), for example, using quantile regression, demonstrated a negative association between inequality and CO_2_. However, no correlation was reported when fixed-effect estimates were considered. Wolde-Rufael and Idowu (2017) did not find any significant short- or long-run link between the two variables. Khan et al. (2018) discovered that income inequality was negatively associated with CO_2_ emissions in India and Pakistan but was positively associated with emissions in Bangladesh. The study by Liu et al. (2019a) indicated that income inequality rises CO_2_ emissions in the short term but has the opposite effect over the long run. Hailemariam et al. (2020) evidenced a negative relationship when top income inequality was used as a measure of inequality, but the opposite result was observed when Gini coefficient was used. Uddin et al. (2020) evidenced for a positive correlation between CO_2_ emissions and inequality between 1870 and 1880, an inverse relationship between 1950 and 2000, and no significant correlation between 1881 and 1949 and 2000 and 2014.

In recent years, the EF has gained attention as a measure for examining the link between environmental degradation and inequality. Unlike CO_2_, which focus solely on pollution in the air, the EF provides a more comprehensive view by considering air, water, and soil. However, relatively few studies examined the association between inequality and the EF. A study conducted by Ekeocha (2021) using Pedroni’s cointegration and quantile regression methods found that inequality was associated with environmental degradation in 46 African countries between 1996 and 2014. According to Kazemzadeh et al. (2021) income inequality had a positive effect on the EF of 25 countries in the period 1970–2016. According to panel quantile regression, income inequality below the 50th quantile had a positive effect on the EF.

According to Khan et al. (2022), income inequality and EFs are positively related, using the standard error method of Driscoll and Kraay. In their study, Langnel et al. (2021) utilized the AMG estimation to examine the effect of natural resources, income inequality, and human capital on the EF of ECOWAS countries. It was determined that while income inequality enhanced environmental quality in three nations, it had the opposite effect in Benin through an increase in the EF. By applying linear and nonlinear ARDL methods to Pakistan from 1972 to 2018, Idrees and Majeed (2022) discovered that environmental degradation is exacerbated by inequality, which has an asymmetric effect on the EF. The FARDL model was utilized by Uzar and Eyuboglu (2023) to investigate the correlation between gini coefficient and the EF in the USA. Inequality in income, they concluded, contributes to an increased EF.

In summary, the research conducted so far does not provide a clear consensus on whether inequality is solely responsible for environmental degradation. Environmental pollution and income inequality are linked positively in some studies, but there has also been evidence of an inverse relationship, varying effects, or insignificance depending on the context and income levels. Therefore, it can be concluded that the effect of inequality on environmental degradation remains a complex and nuanced topic. The existing literature highlights the need for more comprehensive research, considering multiple environmental indicators and examining various regions and time periods. Scholars may contribute to a deeper knowledge of this critical issue by further investigating the link between inequality and environmental degradation, and therefore better inform policies and initiatives aimed at ensuring sustainable and equitable development.

### Energy consumption and environment

There is a substantial body of evidence linking environmental deterioration and energy use. To understand this relationship, several studies have used econometric analyses with CO_2_ or the EF as independent variables. In contrast to the literature on the effects of inequality, both CO_2_ and the EF are widely used as independent variables, with the former being used more frequently than the latter.

Studies using CO_2_ can be classified according to the types of energy considered. More specifically, some studies focus on aggregate energy consumption, whereas others separately consider renewable and non-renewable energies or other forms of energy consumption. Regarding aggregate energy consumption, a number of studies reported a positive relationship with CO_2_ (Hossain 2011; Begum et al. 2015; Alam et al. 2016; Shafiei and Salim 2014; Rahman and Kashem 2017; Ahmad et al. 2019; Muhammad 2019; Adedoyin and Zakari 2020; Rahman 2020; Agboola et al. 2021; Rahman et al. 2021). Regarding the aggregate use of renewable energy consumption, some studies reported a reduction in CO_2_ (Dogan and Seker 2016; Shafiei and Salim 2014; Bhattacharya et al. 2017; Ito 2017; Zoundi 2017; Dong et al. 2018; Charfeddine and Kahia 2019; Zaman et al. 2021; Karaaslan and Çamkaya 2022), whereas others reported an increase (Dogan and Seker 2016; Chen et al 2019; Nathaniel and Iheonu 2019; and Karaaslan and Çamkaya 2022). Regarding specific energy sources, total biomass energy consumption reduced CO_2_ (Kim et al. 2020), and so did nuclear energy consumption (Ozgur et al. 2021). Li and Haneklaus (2022) argued that CO_2_ emissions decrease with increasing clean energy consumption. Conversely, fuel consumption increases CO_2_ emissions (Mohsin et al. 2022). Finally, Salari et al. (2021) investigated the impacts of different types of energy sources on CO_2_, finding that renewable energy decreases CO_2_ emissions, whereas other types of energy increase them.

Several studies utilized the EF as a metric to assess environmental degradation. Charfeddine (2017) discovered that increase in EF is attributable to overall energy consumption. In their study, Alola et al. (2019) found that non-renewable energy usage has a negative impact on environmental quality, while the consumption of renewable energy contributes to the improvement of environmental sustainability and quality. Baz et al. (2020) conducted a study where they utilized asymmetric causality techniques to analyze the asymmetric impact of energy consumption on the EF. It has been determined that energy consumption has a positive influence on the quality of environment.

In their study, Danish et al. (2020) utilized long-run parameter estimators for BRICS countries spanning from 1992 to 2016. Based on their research, they came to the conclusion that using more renewable energy significantly improved environmental quality by lowering the EF. Destek and Sinha (2020) employed second-generation panel data analysis with data from 24 OECD countries between 1980 and 2014. The results revealed that renewable energy consumption led to reduction in the EF. Conversely, the consumption of non-renewable energy sources led to an increase in the EF. A similar observation was made by Nathaniel and Khan (2020), who discovered that the utilization of non-renewable energy sources correlates with a rise in the EF. Nevertheless, the research failed to identify any significant effect of renewable energy on the EF. In their study focused on Turkey, Sharif et al. (2020) found that an increase in renewable energy has a long-run positive impact on reducing the EF. On the other hand, the use of non-renewable energy was found to increase the EF both in the short and long run.

Ali et al. (2021) found that a reduction in EF occurred in countries with varying income groups when there was an increase in renewable energy consumption. Caglar et al. (2021) demonstrated, using the panel ARDL method, that in 10 countries with the most severe environmental degradation, non-renewable energy consumption increases the EF, although renewable energy consumption decreases the EF. According to Nathaniel et al. (2021), renewable energy reduces the EF of the BRICS countries. Furthermore, Ullah et al. (2021) demonstrated that the EF of the world’s top 15 renewable energy-consuming economies decreased between 1996 and 2018. Shahzad et al. (2021) demonstrated, using QARDL and quantile Granger causality tests, that fossil fuel energy consumption in the USA increases the EF.

Through the utilization of the CS-ARDL methodology, Sharma et al. (2021) reported that the consumption of renewable energy results in a reduction of the EF in eight Asian developing countries. Usman and Makhdum (2021) conducted a panel data analysis for BRICS-T countries between 1990 and 2018 and discovered that non-renewable energy consumption increases the EF, whereas renewable energy consumption decreases it. Abid et al. (2022) discovered a negative and significant link between renewable energy consumption and the EF in Saudi Arabia from 1980 to 2017. Adekoya et al. (2022) discovered that nonrenewable energy consumption has a negative impact on the environment in both net-oil exporter and net-oil importer nations. Conversely, renewable energy consumption reduces the EF of net oil importers.

Gupta et al. (2022) concluded that increased energy use increases Bangladesh’s EF. Utilizing datasets from 120 countries, Li et al. (2022a, b) discovered that the EF increases with the spread of urbanization, whereas it initially decreases with the utilization of renewable energy sources. Xu et al. (2022) discovered that renewable energy decreases the EF in the long run. Huang et al. (2022) discovered from their analysis of the E7 countries from 1995 to 2018 using the second-generation method that renewable energy reduces their EF. Liu et al. (2022a, b) discovered that energy use in Pakistan, which is primarily based on fossil fuels, harms environmental quality by increasing the EF. In contrast to the assertion made by Yang et al. (2021) that energy consumption exacerbates environmental problems through an augmentation of the EF, Miao et al. (2022) demonstrated that renewable energy technology reduces the EF. A negative correlation was discovered between biomass energy consumption and the EF by Yasmeen et al. (2022), suggesting that biomass energy consumption has a negative impact on the environment.

Overall, the literature based on the EF, which employs various methodologies and covers a wide range of estimation samples, consistently indicates that increasing the use of renewable energy has a positive impact on environmental quality, as measured by the size of the EF, whereas reliance on fossil fuels has an adverse impact. Research using the load capacity factor derived from EF, e.g., Pata and Balsalobre-Lorente (2022) and Shang et al. (2022), produced qualitatively similar results.

### General evaluation and literature gap

As revealed by the literature review, there is no agreement on the presence of the EKC hypothesis. The analysis of this validity using panel threshold models is quite rare, with Aydin et al. (2019) selecting GDP as the threshold variable for EU countries. Given the global impact of the climate crisis, expanding the country sample can enable scholars to discuss more comprehensive policy implications for the world, rather than focusing on a specific group of countries. Detecting GDP as a threshold variable in this study enables the evaluation of the EKC relationship without introducing the squared and cube of GDP into the model. Inclusion of those variables might lead to a collinearity problem and biased estimates, undermining the credibility of policy implications (Sirag et al. 2017; Aydin et al. 2019).^1^ Furthermore, the use of GDP as a threshold variable provides identification of the potential effects of economic growth and other key determinants on environmental degradation under the different phases of economies. By analyzing countries with varying levels of GDP, researchers can identify how different economic factors interact with climate change and contribute to policy recommendations that are tailored to each country’s unique circumstances. Additionally, focusing on a broader country sample allows for a more accurate assessment of the global nature of the climate crisis and emphasizes the need for international cooperation and coordination in addressing this urgent issue.

It is also worth mentioning that the increase in income inequality has become another important problem on a global scale. Hence, this study also aims to contribute to the existing literature by analyzing the presence of EKC in terms of the link between income inequality and EF in a nonlinear framework.

## Data and model

This study examines the relationship between EF, economic growth, income inequality, consumption of renewable and nonrenewable energy, and globalization in 49 countries from 1995 to 2018. Within the scope of the study, the linear form of the estimated panel data model follows the standard structure of a panel data model:

1 $${lnEF}_{it}={\beta }_{0}+{\beta }_{1}{lnGDP}_{it}{+{\beta }_{2}{lnII}_{it}+\beta }_{3}{lnREC}_{it}{+\beta }_{4}{lnNREC}_{it}+{\beta }_{5}{lnKOF}_{it}+{\varepsilon }_{it}$$

where ${lnEF}_{it}$ represents the natural log of EF in gha per capita; ${lnII}_{it}$ represents the natural log of Gini coefficient based on disposable income $; {lnGDP}_{it}$ represents the natural log of real GDP per capita; ${lnREC}_{it}$ represents the natural log of renewable energy consumption;${lnNREC}_{it}$; represents the natural log of nonrenewable energy consumption $;{\text{and}} {lnKOF}_{it}$ represents the natural log of the KOF globalization index. As the model is written in double-log form, parameter estimates indicate elasticities. The countries in the estimation sample are selected based on the availability of the data. The detailed information on the units and sources of the data is presented in Table 1.

**Table 1:** Summary table for the variables

Variable name	Variable name abbreviation	Unit	Source
Ecological footprint	${EF}_{it}$	Gha per capita	Global Footprint Network (2022)
Gross domestic product per capita	${GDP}_{it}$	Constant 2015 US dollars	Refinitiv Eikon Datastream (2022)
Income inequality	${lnII}_{it}$	Gini coefficient based on disposable income	SWIID (2022)
Renewable energy consumption	${REC}_{it}$	Terawatt hour	Refinitiv Eikon Datastream (2022)
Nonrenewable energy consumption	${NREC}_{it}$	Terawatt hour	Refinitiv Eikon Datastream (2022)
Globalization	${KOF}_{it}$	KOF globalization index	KOF Swiss Economic Institute (2022)

The EF is a metric measuring the demand for nature generated by competing agents, such as individuals, products, and populations that require natural resources for consumption and waste disposal. The EF is calculated based on six categories: farmland, grazing land, fishing grounds, developed land, forest area, and land carbon demand, which are added together to determine the overall footprint. The computation is based on the amount of natural sources that humans have and the amount they use, both in global hectares (Global Footprint Network 2023).

To understand the factors contributing to environmental degradation, it is critical to consider economic growth because studies have shown that it is responsible for environmental damage (e.g., Alola et al. 2019; Danish and Wang 2019; Nathaniel and Khan 2020). For this study, GDP per capita is computed by dividing GDP by population, and it is expected to have a positive relationship with EF.

Income inequality refers to the gap between the wealthy and the poor, and describes the unequal distribution of assets, income, or wealth among individuals or groups (OECD 2023). There are various perspectives on how inequality affects the environment. According to one viewpoint, it may lead to overuse of natural resources by the poor to survive in harsh living conditions, resulting in a decline in environmental protection. Another point of view holds that as income inequality increases, the marginal propensity to emit declines. Moreover, it is worth noting that there may be a positive correlation between the increase in income inequality and the corresponding rise in energy demand and environmental pollution. In addition to these viewpoints, some studies yielded results indicating the lack of a statistically significant relationship (e.g., Wolde-Rufael and Idowu 2017). The lack of agreement on the connection between income inequality and environmental problems implies that its impact could be positive, negative, or insignificant.

Renewable energy sources, which include bioenergy, geothermal energy, hydrogen, hydropower, marine energy, solar energy, and wind energy, are derived from naturally replenished resources (Office of Energy Efficiency and Renewable Energy 2023). Renewable energy is crucial to avoid environmental degradation (UN 2023), so it is expected to reduce the EF. Nonrenewable energy consumption consists mostly of fossil fuels, i.e., oil, coal, and natural gas, and is thus predicted to have a positive impact on the EF.

As an indicator of globalization, Dreher (2006) introduced the KOF globalization index. Contradictory findings have been reported in numerous studies that have attempted to determine whether globalization has an effect on the EF. Saud et al. (2020) and Wang et al. (2020) both documented a negative connection between KOF globalization indices and the EF. On the contrary, several investigations have concluded that globalization does not have an important impact on the EF (Ahmed et al. 2019) or have positive effect (Sabir and Gorus 2019; Pata 2021). Farooq et al. (2022) discovered that economic globalization degrades the environment. An identical finding is corroborated by Ahmed et al. (2021). The anticipated impact of this variable may be positive or negative, given the absence of consensus regarding the effects of globalization.

**Fig. 1:**
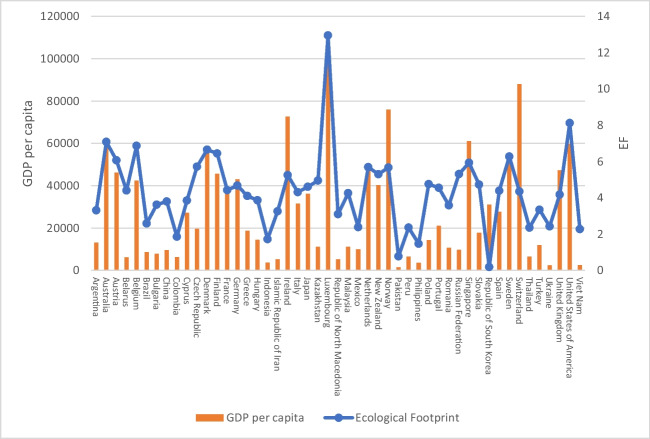
GDP per capita and EF in 2018. Source: Global Footprint Network (2022); Refinitiv Eikon Datastream (2022)

The descriptive statistics of the variables are provided in Table 2. Figure 1, which illustrates the per capita EF and GDP in 2018, provides additional insight into the relationship between variables. It is evident that there exists a positive correlation between EF and GDP per capita, with the exception of a few countries. Consequently, nations characterized by a greater GDP per capita generally exhibit larger EFs. This association is influenced by several factors, including increased energy consumption and reliance on fossil fuels (Alola et al. 2019). This suggests the potential for a compromise to be made between environmental quality and economic growth. As a result, the main objective of this research is to examine this connection.

**Table 2:** Descriptive statistics

Variable	Mean	Standard deviation	Minimum	Maximum
${lnEF}_{it}$	1.3271	0.7485	− 2.7999	2.8750
${lnGDP}_{it}$	9.5403	1.1663	6.5924	11.6295
${lnII}_{it}$	3.4929	0.2085	3.0864	3.9926
${lnREC}_{it}$	2.2784	1.1665	− 1.1227	4.1763
${lnNREC}_{it}$	4.4888	0.9437	0.7566	6.4531
${lnKOF}_{it}$	4.2653	0.1930	3.4744	4.5078

## Methodology

This study employs the panel threshold model that Hansen (1999) proposed to examine the nonlinear impacts of the explanatory variables on the EF. This model allows for more precise estimates in the presence of data heterogeneity and structural breaks that differ across countries. Accordingly, the fixed-effect version of the panel threshold model is presented as follows:

2 $${y}_{it}=\mu +{X}_{it}\left({q}_{it}<\gamma \right){\beta }_{1}+{X}_{it}\left({q}_{it}\ge \gamma \right){\beta }_{2}+{{u}_{i}+e}_{it}$$

where ${q}_{it}$ is the threshold variable; $\gamma$ shows the threshold parameter that divides the model into two separate regimes; ${\beta }_{1}$ and ${\beta }_{2}$ are the coefficients of the two separate regimes; ${u}_{i}$ shows the individual effect; and ${e}_{it}$ refers to the disturbance term.

Equation (2) can be rewritten as follows:

3 $${y}_{it}=\mu +{X}_{it}\left({q}_{it},\gamma \right)\beta +{{u}_{i}+e}_{it}$$

4 $${X}_{it}\left({q}_{it},\gamma \right)=\{{X}_{it}I\left({q}_{it}<\gamma \right) {X}_{it}I\left({q}_{it}\ge \gamma \right)$$

In Eq. (3), the parameters of the threshold model $\beta$ estimated with the ordinary least squares are formulated as follows:

5 $$\widehat{\beta }={\left\{{X}^{*}{\left(\gamma \right)}{\prime}{X}^{*}(\gamma )\right\}}^{-1}\left\{{X}^{*}{\left(\gamma \right)}{\prime}{y}^{*}\right\}$$

The residual sum of squares (RSS) is equal to ${\widehat{e}}^{{*}{\prime}}{\widehat{e}}^{*}$. Here, $\gamma$ can be estimated by limiting the range ($\gamma , \underset{\_}{\gamma )}$, which is the quantile of ${q}_{it}$, while $\widehat{\gamma }$ minimizes the RSS, where $\widehat{\gamma }=argarg {S}_{1}( \gamma )$. If $\gamma$ is known, the model is identical to the standard linear model, whereas if it is unknown, the distribution of the $\gamma$ estimator becomes nonstandard. Hansen (1999) showed that $\widehat{\gamma }$ is a consistent estimator, so the optimal method for testing $\gamma$ = ${\gamma }_{0}$ is to form a confidence interval using the no-rejection region approach with the likelihood ratio (LR) statistic as follows:

$${LR}_{1}\left(\gamma \right)=\frac{\left\{{LR}_{1}\left(\gamma \right)-{LR}_{1}\left(\widehat{\gamma }\right)\right\}}{{\widehat{\sigma }}^{2}} Pr\to \xi$$

6 $$PrPr \left(x<\xi \right) ={(1-{e}^{\frac{-x}{2}})}^{2}$$

The *α* quantile is calculated by the inverse function of Eq. (7):

7 $$c\left(\alpha \right)=-2log(1-\sqrt{1-\alpha })$$

The null hypothesis is rejected if ${LR}_{1}\left({\gamma }_{0}\right)$ is higher than $c\left(\alpha \right)$. The null hypothesis is equal to ${H}_{o}:{\beta }_{1}={\beta }_{2}$ and ${H}_{a}:{\beta }_{1}\ne {\beta }_{2}$, while the *F* statistic is ${F}_{1}=\frac{({S}_{0}-{S}_{1})}{{\widehat{\sigma }}^{2}}$. Under the null hypothesis, the threshold $\gamma$ has not been identified and ${F}_{1}$ does not have a standard asymptotic distribution. Hansen (1999) therefore suggested the following bootstrapping method.

First, the model is fitted under the alternative hypothesis to obtain the residual $${\widehat{e}}_{it}^{*}$$. Second, the replacement technique is used to perform cluster resampling to obtain the new residual $${v}_{it}^{*}$$. Third, the new series are generated under the alternative hypothesis: $${y}_{it}^{*}={X}_{it}^{*}\beta +{v}_{it}^{*}$$. Fourth, the model is fitted under both null and alternative hypotheses and the *F* statistic is computed. Finally, the same four steps are repeated B times with the probability that *F* is Pr = *I*(*F* > $${F}_{1})$$.

If multiple thresholds exist, the double-threshold model is used:

8 $${y}_{it}=\mu +{X}_{it}\left({q}_{it}<{\gamma }_{1}\right){\beta }_{1}+{X}_{it}\left({{\gamma }_{1}\le q}_{it}<{\gamma }_{2}\right){\beta }_{2}+{{{X}_{it}({q}_{it}\ge {\gamma }_{2}){\beta }_{3}+u}_{i}+e}_{it}$$

where the thresholds ${\gamma }_{1}$ and ${\gamma }_{2}$ split into three regimes with the coefficients ${\beta }_{1}$, ${\beta }_{2},$ and ${\beta }_{3}$. The thresholds are estimated by the following three steps:

- 1. The single threshold model is fitted to get ${\gamma }_{1}$ and RSS ${S}_{1}({\widehat{\gamma }}_{1})$.
- 2. Given ${\widehat{\gamma }}_{1}$, the second threshold and its confidence interval can be derived as follows:
  $${\widehat{\gamma }}_{2}^{r}=argmin\left\{{S}_{2}^{r}({\gamma }_{2})\right\}$$
  $${S}_{2}^{r}=S\left\{min({\widehat{\gamma }}_{1},{\gamma }_{2})max({\widehat{\gamma }}_{1},{\gamma }_{2})\right\}$$
  $${LR}_{2}^{r}({\gamma }_{2})=\frac{\left\{{S}_{2}^{r}\left({\gamma }_{2}\right)-{S}_{2}^{r}({\widehat{\gamma }}_{2}^{r})\right\}}{{\widehat{\sigma }}_{22}^{2}}$$
- 3. Since ${\widehat{\gamma }}_{2}^{r}$ is efficient but ${\widehat{\gamma }}_{1}^{r}$ is inefficient, the first threshold is re-estimated as follows:
  $${\widehat{\gamma }}_{1}^{r}=argmin\left\{{S}_{1}^{r}({\gamma }_{1})\right\}$$
  $${S}_{1}^{r}=S\left\{min({\gamma }_{1},{\widehat{\gamma }}_{2})max({\gamma }_{1},{\widehat{\gamma }}_{2})\right\}$$
  $${LR}_{1}^{r}({\gamma }_{1})=\frac{\left\{{S}_{1}^{r}\left({\gamma }_{1}\right)-{S}_{1}^{r}({\widehat{\gamma }}_{1}^{r})\right\}}{{\widehat{\sigma }}_{21}^{2}}$$

If ${H}_{o}$ in the single-threshold model is rejected, the existence of double-threshold model should be examined. In that case, the null hypothesis of the single-threshold model is tested against the alternative hypothesis of the double-threshold model. In this case, the *F* statistic is equal to ${F}_{2}=\frac{\left\{{S}_{1}\left({\widehat{\gamma }}_{1}\right)-{S}_{2}^{r}({\widehat{\gamma }}_{2}^{r})\right\}}{{\widehat{\sigma }}_{22}^{2}}$. The bootstrapping procedure in the double-threshold model is identical to the single-threshold model. In the third step, a new series under the null hypothesis is generated, where ${y}_{it}^{*}={X}_{it}^{*}{\beta }_{S}+{v}_{it}^{*}$. Similar steps need to be repeated for models with more than two threshold parameters using the same testing procedure. In the final step, after determining the optimum number of breaks and the threshold value, the model is estimated with the fixed-effect estimator defined in Eq. (2).

## Empirical results

### Second-generation panel data estimates

This section begins by reporting the test for cross-sectional dependence using the CD test (Pesaran 2004). Based on the results presented in Table 3, the null hypothesis of cross-sectional dependency is rejected at a significance level of 1%. This suggests that the variables in question exhibit cross-sectional dependence. This implies that a shock occurring in one of the analyzed countries has the potential to spread to the others. The homogeneity test results for the slope coefficients, conducted by Pesaran and Yamagata (2008), are presented in panel b of Table 3. This test is used to evaluate the homogeneity of the slope coefficients. Pesaran and Yamagata (2008) test results reveal the heterogeneity in the slope coefficients.

**Table 3:** Cross-sectional dependence and homogeneity test results

a. Cross-section dependence test		
	Statistic	*p*-value
LM	1411	0.0000***
LM adj*	1.729	0.0838*
LM CD*	6.917	0.0000***
b. Slope homogeneity test		
	$\widetilde{\Delta }$	${\widetilde{\Delta }}_{adj}$
Test statistic	20.665	24.554
*p*-value	0.000***	0.000***

^*^, **, and *** denote statistical significance at 10%, 5%, and 1% levels, respectively

The CIPS panel unit root test proposed by Pesaran (2007) is employed to determine the order of integration of variables after conducting checks for heterogeneity and cross-sectional dependency. The results presented in Table 4 indicate that all the variables are integrated of order 1, which is referred to as *I*(1). Upon verifying that the variables are degree of integration of the variables, the long-run relationship is examined through the use of panel cointegration test developed by Westerlund (2007), which considers cross-sectional dependency. According to the information presented in Table 5, there is strong statistical evidence supporting the long-term relationship, with *p*-values that are significant at the 1% level.

**Table 4:** Pesaran (2007) CIPS panel unit root tests results

	*lnEF*_*it*_	*lnII*_*it*_	*lnGDP*_*it*_	*lnREC*_*it*_	*lnNREC*_*it*_	*lnKOF*_*it*_
Level	− 1.522	− 1.546	− 2.014	− 2.144	− 1.211	− 2.682
First diff	− 4.972***	− 3.062***	− 2.800***	− 4.603***	− 4.309***	− 5.005***

^***^ denotes significance at 1% level. Critical values are − 2.04, − 2.011, and − 2.23 for 10%, 5%, and 1% significance levels, respectively

The CCEMG estimator developed by Pesaran (2006) and the AMG estimator proposed by Eberhardt and Bond (2009) are utilized to estimate the long-run determinants of EF, given that all the variables are integrated of order 1 and there exists a cointegration relationship between the variables. The AMG estimator results indicate that the parameter estimates for the natural log of GDP ($${lnGDP}_{it}$$), renewable energy consumption ($${lnREC}_{it}$$), and nonrenewable energy consumption ($${lnNREC}_{it}$$) are statistically significant. However, there is no significant effect of income inequality ($${lnII}_{it})$$ and globalization ($${lnKOF}_{it})$$ on the EF.

The coefficient for ${lnGDP}_{it}$ is estimated to be 0.4528. This suggests that a 1% increase in GDP leads to a 0.4528% increase in the EF. The coefficient for the ${lnREC}_{it}$ is estimated as − 0.0745. This suggests that a 1% increase in renewable energy consumption leads to a decrease in the EF by 0.0745% (Table 6). The parameter for ${lnNREC}_{it}$ is estimated to be 0.3729. This means that a 1% increase in primary energy consumption will result in a 0.3729% increase in the EF. The positive and significant impact of GDP and nonrenewable energy consumption, and the negative and significant effect of renewable energy consumption on the EF are also supported by the results of the CCEMG estimator. Similarly, income inequality ${lnII}_{it}$ has no significant effect on the EF. The only exception is that the parameter sign of the ${lnKOF}_{it}$ is positive, contrary to the AMG estimator, and statistically significant at the 10% level. The coefficient of ${lnKOF}_{it}$ is estimated as 0.4667, indicating that a 1% increase in the globalization index increases the EF by 0.4667%.

**Table 5:** Westerlund (2007) cointegration test results

Statistic	Value	*Z* value	*P-v*alue	Robust *P-*value
*G*_*t*_	− 3.692	− 10.279	0.000	0.000***
*G*_*a*_	− 6.367	4.883	1.000	0.000***
*P*_*t*_	− 23.441	− 8.404	0.000	0.000***
*P*_*a*_	− 7.993	− 0.034	0.514	0.000***

In summary, the findings suggest that the EF is increased by GDP and nonrenewable energy consumption, while it is reduced by renewable energy consumption. Several previous studies have confirmed that the environmental degradation can be reduced by increasing the use of renewable energy (Danish et al. 2020; Destek and Sinha 2020; Sharif et al. 2020; Ali et al. 2021; Caglar et al. 2021; Nathaniel et al. 2021; Ullah et al. 2021; Sharma et al. 2021; Usman and Makhdum 2021; Abid et al. 2022; Huang et al. 2022; Miao et al. 2022; Zeng et al. 2023; Kartal et al. 2023). The findings that higher energy consumption increases the EF are aligned with the studies conducted by Charfeddine (2017), Baz et al. (2020), Liu et al. (2022a, b), and Yang et al. (2021). Previous research, including this study, has shown that economic growth has a positive impact on the EF (Alola et al. 2019; Danish and Wang 2019; Nathaniel and Khan 2020; Sharif et al. 2020; Ali et al. 2021; Nathaniel et al. 2021; Ullah et al. 2021; Usman et al. 2021; Yang et al. 2021; Çakmak and Acar 2022; Liu et al. 2022a, b; Miao et al. 2022; Destek et al. 2023b; Pata et al. 2024b).

The research findings on the impact of income inequality and the KOF index on the EF are inconclusive due to variations in results based on the estimation method used. The conclusion that income inequality has a negligible impact on environmental degradation is corroborated by the studies conducted by Clement and Meunie (2010), Kasuga and Takaya (2017), Wolde-Rufael and Idowu (2017), Chen et al. (2020), Wu and Xie (2020), and Zhao et al. (2021). This indicates that redistributing income as a strategy to address environmental degradation may not produce effective outcomes. The finding that globalization has an insignificant effect on the EF is also supported by Ahmed et al. (2019). The analysis indicates that implementing measures to address globalization may not yield significant environmental benefits for the countries under consideration.

It is important to acknowledge that the long-run estimator’s results depend on the assumption of linearity in the relationship between the variables and the EF. However, these results may be subject to certain limitations and should be interpreted with caution.

### Panel threshold regression estimation results

In order to further investigate this relationship, we employ the panel threshold model in the error correction form, as proposed by Ho and Chiu (2001), Wang (2012), and Wang and Lee (2018). This approach allows us to account for potential threshold effects and estimate the long-run dynamics between the variables. The empirical results of the panel threshold model are presented in Table 6 and 7. As explained in the “Methodology” section, the first step of the estimation is determining the transition variable, ${q}_{it}$, assumed to have a threshold effect on the dependent variable. Following Odhiambo (2020), Huang and Duan (2020), Lee et al. (2022), and Asiedu et al. (2022), two potential threshold variables from among the independent variables are considered in the the panel error correction model: the log first difference of Gini coefficient ${\Delta lnII}_{it}$ and the log first difference of GDP per capita ${\Delta lnGDP}_{it}$ (economic growth).

**Table 6:** AMG and CCEMG long-run estimators test results

Dependent variable:${lnEF}_{it}$	AMG estimator		CCE estimator	
Regressors	Coefficient	*P* value	Coefficient	*P* value
${lnGDP}_{it}$	0.4528	0.000***	0.5845	0.000***
${lnII}_{it}$	0.1958	0.553	0.2734	0.589
${lnREC}_{it}$	− 0.0745	0.029**	− 0.1025	0.031**
${lnNREC}_{it}$	0.3729	0.000***	0.2330	0.013**
${lnKOF}_{it}$	− 0.1207	0.520	0.4667	0.059*
Constant	− 4.7094	0.002***	2.7042	0.574

^*^, **, and *** denote the statistical significance at 10%, 5%, and 1% levels, respectively

To determine the optimal number of thresholds and their optimum values ($\gamma )$, the approach proposed by Hansen (1999) is adopted, which employs the bootstrap technique to approximate the *F*-statistics. The bootstrap process is repeated for 1000 iterations to detect any panel threshold effects. Table 7 displays the empirical results of the test for the two alternative threshold. The findings suggest that when income inequality is used as the threshold variable, there is evidence to reject the null hypothesis of no threshold. This is supported by the fact that the computed *F* statistics are lower than the critical value. Therefore, the results do not support a nonlinear relationship between the income inequality and EF.

**Table 7:**
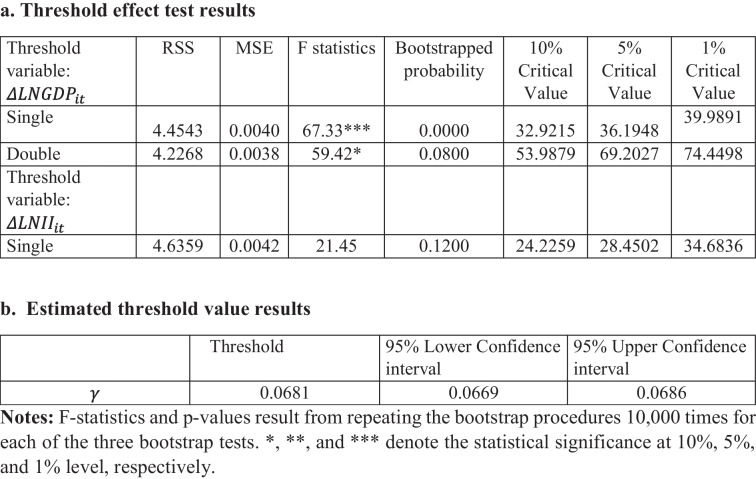
Threshold test results

Regarding the test results using the economic growth (${\Delta lnGDP}_{it}$) threshold variable, the null hypothesis of no threshold is rejected in favor of a single threshold. The *F* statistic is significant at the 1% level of significance, with a bootstrapped *p*-value of 0.00. The results, however, indicate that the presence of two thresholds is rejected at the 5% significance level, as indicated by the bootstrapped *p*-value of 0.08 *F* statistics. The higher value of the calculated *F* statistic for the single threshold model as compared to the double-threshold model provides additional support for the selection of a single threshold.

Therefore, the test result reveals the presence of a single-threshold value estimated to be 0.0681 and indicates the presence of two distinct regimes based on the optimal value of economic growth: the lower regime ($\gamma <0.0681$) and upper regime ($\gamma \ge 0.0681$). For the remainder of the analysis, ${\Delta lnGDP}_{it}$ is employed as the transition variable in the panel threshold error correction estimates.

As a result of the nonlinearity determined between EF and ${\Delta lnGDP}_{it}$, Table 8 shows the estimation of the two-regime panel threshold error correction model. Before proceeding to the interpretation of the variables, it is worth mentioning that the error correction terms are negative and significant in both the lower and upper growth regimes. This suggests that any short-run departure from long-run equilibrium is corrected, and the model has been correctly specified.

The coefficient estimates derived from the panel threshold model offer valuable insights into the link between economic growth and the EF across regimes. More specifically, the results reveal that economic growth has a significantly positive impact on the EF in both regimes (see Table 7). The lower regime exhibits an estimated economic growth coefficient of 0.663, which signifies that a 1% increase in ${\Delta lnGDP}_{it}$ corresponds to a 0.663% increase in the EF (Table 8). Likewise, a 1% increase in ${\Delta lnGDP}_{it}$ is associated with a 0.558% increase in the EF, as indicated by the estimated coefficient for economic growth in the upper regime.

**Table 8:** Panel threshold error correction estimation results (threshold variable: $\Delta {lnGDP}_{it}$)

Dependent variable:${\Delta lnEF}_{it}$	Coef	Std. err	*t*-statistics	Probability	[95% conf. interval]	
$$\Delta {lnGDP}_{it}$$						
Lower regime	0.663***	0.081	8.200	0.000	0.504	0.821
Upper regime	0.558***	0.149	3.740	0.000	0.265	0.851
$${\Delta lnII}_{it}$$						
Lower regime	− 0.322	0.224	− 1.440	0.150	− 0.760	0.117
Upper regime	− 0.083	0.623	− 0.130	0.894	− 1.306	1.139
$$\Delta {lnREC}_{it}$$						
Lower regime	− 0.034*	0.019	− 1.750	0.080	− 0.072	0.004
Upper regime	− 0.177**	0.076	− 2.330	0.020	− 0.326	− 0.028
$$\Delta {lnNREC}_{it}$$						
Lower regime	0.377***	0.047	8.060	0.000	0.285	0.469
Upper regime	0.426***	0.144	2.960	0.003	0.143	0.708
$${\Delta lnKOF}_{it}$$						
Lower regime	− 0.296**	0.121	− 2.440	0.015	− 0.534	− 0.058
Upper regime	− 0.048	0.403	− 0.120	0.905	− 0.839	0.743
$${ECM}_{t-1}$$						
Lower regime	− 0.350***	0.024	− 14.750	0.000	− 0.397	− 0.303
Upper regime	− 0.424***	0.024	− 17.590	0.000	− 0.471	− 0.376

^*^, **, and *** denote the statistical significance at 10%, 5%, and 1% levels, respectively

The EKC hypothesis is not supported by evidence on positive and statistically significant parameter estimates for economic growth in both regimes. The EKC theory proposes an inverted U-shaped relationship between economic growth and environmental degradation, in which environmental impacts initially increase but then decrease as economies progress. However, regardless of the regime, the results show that economic growth has a significant positive impact on the EF. This finding is consistent with Sirag et al. (2017), Aydin et al. (2019), and Wang et al. (2022), but differs from Wu and Liu (2020), Simionescu (2021), and Wang et al. (2023).

Regarding the Gini coefficient ($\Delta {lnII}_{it})$, the parameter estimates are found to be statistically insignificant for both the lower and upper regimes, indicating that changes in the Gini coefficient have no significant impact on the environmental pollution. Regarding globalization, no significant effect of the globalization index on the EF is evidenced in the upper regime, as the economic growth exceeds the optimum threshold ($\gamma \ge 0.0681$). On the contrary, in the lower growth regime, the estimated parameter for the KOF globalization index is − 0.296, which is statistically significant at the 5% level. This suggests that a 1% increase in globalization is associated with a 0.296% reduction in the EF. Hence, it can be inferred that as nations attain greater economic growth, the effects of globalization become less significant. Wang et al. (2020) and Saud et al. (2020) have similarly revealed a negative effect of globalization on the EF, which is consistent with these findings.

The panel threshold analysis reveals significant findings concerning the parameter estimates of nonrenewable energy consumption in both regimes (see Table 7). The parameter estimate for nonrenewable energy consumption in the lower regime is positive and significant, implying that a 1% increase in the use of nonrenewable energy increases the EF by 0.377%. In the upper regime, the parameter estimate for nonrenewable energy consumption is slightly higher at 0.426. This indicates that increased use of non-renewable energy sources considerably leads to an increase in the EF in both lower and upper growth regimes, but this effect is more pronounced when the economy exceeds the optimum threshold growth rate ($\gamma \ge 0.0681$). Similar to this study’s findings, Alola et al. (2019), Destek and Sinha (2020), Nathaniel and Khan (2020), Sharif et al. (2020), Caglar et al. (2021), Shahzad et al. (2021), Usman and Makhdum (2021), Adekoya et al. (2022), and Li et al. (2022a, b) have reported the escalating impact of nonrenewable energy on environmental pollution.

The coefficient estimates for renewable energy consumption are statistically significant in both regimes. In the lower regime, the estimated parameter is − 0.034, indicating that a 1% increase in renewable energy consumption decreases the EF by 0.034%. Notably, the impact of renewable energy consumption becomes even more pronounced in the upper regime, with an estimated coefficient of − 0.177. This indicates that a 1% increase in renewable energy consumption decreases the EF three times more than in the lower regime. Hence, it can be concluded that renewable energy sources benefit the environment more for economies that have surpassed a certain economic growth threshold. This finding is aligned with those of Ullah et al. (2021) and Chen et al. (2022).

Overall, the analysis in the present study provides empirical evidence that economic growth and nonrenewable energy consumption both significantly increase the EF, whereas renewable energy consumption significantly reduces it. The impact of renewable energy consumption is more pronounced in the upper than lower economic growth regimes. As far as panel threshold estimates indicate, income inequality has no significant impact on the EF, as evidenced by the insignificant coefficient estimates in both upper and lower growth regimes.

## Conclusions

This paper examines the determinants of the ecological footprint for a panel of 49 countries from 1995 to 2018. The analysis provides empirical evidence on the impact of economic growth, income inequality, renewable energy consumption, nonrenewable energy consumption, and globalization on the ecological footprint. The study examines the relationship between the variables using both second-generation panel data and panel threshold error correction models.

The findings regarding the AMG and CCEMG long-run parameter estimates reveal that economic growth and nonrenewable energy consumption both significantly increase the ecological footprint, whereas renewable energy consumption significantly reduces it. However, panel threshold model estimates that used economic growth as the transition variable suggests that the impact of renewable energy consumption on the ecological footprint is nonlinear as the impact of this variable more pronounced in the upper than lower growth regime. That is, once economies exceed a certain level of economic growth, the use of renewable energy sources has a greater beneficial effect on the environment. The estimation sample used in the present study also provides no support for the EKC hypothesis. These findings suggest that the assumed monotonic relationship between economic growth and environmental degradation, as proposed by the EKC hypothesis, does not hold true. Rather, the link between economic growth and environmental pollution is more intricate and multifaceted.

### Policy implications

The empirical evidence of the study has important policy implications. The findings from econometric analysis imply that policies promoting renewable energy consumption can reduce the ecological footprint, but particularly in high income countries. Furthermore, the results suggest that income inequality has no significant impact on the ecological footprint. This suggests that policies targeting income inequality reduction may not necessarily reduce a country’s ecological footprint. Policymakers may therefore shift their focus toward other factors that have a more significant impact, such as investing in clean energy technologies and transitioning to renewable energy sources.

Given that the connection between economic growth and environmental degradation is more intricate and multifaceted than previously documented, policymakers might consider this complexity when formulating strategies to mitigate the ecological footprint. For example, policymakers may consider implementing policies leading to reduction in the GDP growth. However, it is important to acknowledge that increasing incomes play a crucial role in the adoption of clean energy technologies and the promotion of renewable energy consumption. Lower economic growth might impede countries from investing in clean energy technologies or hinder their ability to transition to renewable energy sources. Overlooking this complex relationship may result in unfavorable outcomes or unintended consequences. To make effective policy decisions in this context, it is vital for policymakers to understand the interplay between economic growth and environmental degradation in a comprehensive manner.

### Future research suggestion

The paper ends with two directions for future research. In this study, it has been endeavored to analyze the largest possible country sample due to data availability. An analogous analysis can be performed for a selection of specific countries, such as OECD and G7 countries, in order to draw conclusions regarding the effectiveness of environmental policies. Moreover, it may be worth considering the replacement of the ecological footprint with the load capacity factor as a comprehensive indicator of environmental problems, enabling an examination of environmental issues.

## Data Availability

The datasets analyzed during the current study are available from the corresponding author on reasonable request.

## Abbreviations

AMG: Augmented mean group
ARDL: Autoregressive distributed lag
BP: British Petroleum
BRICS: Brazil, Russia, India, China, and South Africa
BRICS-T: Brazil, Russia, India, China, South Africa, and Turkey
CCEs: Central and Eastern European Countries
CCEMG: Common correlated effect mean group
CD: Cross-sectional dependence
CO_2_: Carbon dioxide
CS-ARDL: Cross-sectionally augmented autoregressive distributed Lag
DOLS: Dynamic ordinary least squares
ECOWAS: Economic Community of West African States
EF: Ecological footprint
EKC: Environmental Kuznets curve
EU: European Union
FARDL: Fuzzy autoregressive distributed lag
FMOLS: Fully modified ordinary least squares
GDP: Gross domestic product
GHA: Global hectare
GHG: Greenhouse gases
GMM: Generalized method of moments
II: Income inequality
LM: Likelihood ratio
MENA: Middle East and North Africa
NREC: Nonrenewable energy consumption
OECD: Organization for Economic Co-operation and Development
OLS: Ordinary least squares
PSTR: Panel smooth transition regression
QARDL: Quantile autoregressive distributed lag
REC: Renewable energy consumption
RSS: Residual sum of squares
STIRPAT: Stochastic impacts by regression on population, affluence, and technology
SWIID: Standardized World Income Inequality Database
UN: United Nations

## Footnotes

1 In this study, GDP square is initially included in the linear model. However, the results suggest the presence of high collinearity between lnGDP and lnGDP^2^, with a variance inflation factor of 262.61, remarkably higher than the critical value of 10. Hence, this variable is excluded from the linear estimates. This evidence also provides another justification for the use of the panel threshold model.
